# Complement System in Cutaneous Squamous Cell Carcinoma

**DOI:** 10.3390/ijms20143550

**Published:** 2019-07-19

**Authors:** Pilvi Riihilä, Liisa Nissinen, Jaakko Knuutila, Pegah Rahmati Nezhad, Kristina Viiklepp, Veli-Matti Kähäri

**Affiliations:** 1 Department of Dermatology, University of Turku and Turku University Hospital, Hämeentie 11 TE6, FI-20520 Turku, Finland; 2 The Western Cancer Centre of the Cancer Center Finland (FICAN West), University of Turku and Turku University Hospital, Kiinamyllynkatu 10, FI-20520 Turku, Finland

**Keywords:** complement, skin cancer, squamous cell carcinoma

## Abstract

Epidermal keratinocyte-derived cutaneous squamous cell carcinoma (cSCC) is the most common metastatic skin cancer with high mortality rates in the advanced stage. Chronic inflammation is a recognized risk factor for cSCC progression and the complement system, as a part of innate immunity, belongs to the microenvironment of tumors. The complement system is a double-edged sword in cancer, since complement activation is involved in anti-tumor cytotoxicity and immune responses, but it also promotes cancer progression directly and indirectly. Recently, the role of several complement components and inhibitors in the regulation of progression of cSCC has been shown. In this review, we will discuss the role of complement system components and inhibitors as biomarkers and potential new targets for therapeutic intervention in cSCC.

## 1. Introduction

Keratinocyte-derived non-melanoma skin cancers (NMSC) are the most common human malignancies and their incidence is increasing worldwide [1]. Cutaneous squamous cell carcinomas (cSCC) represent approximately 20% of NMSCs and they are mainly responsible for deaths in NMSC. The major risk factors for cSCC are long-term exposure to solar ultraviolet (UV) radiation, chronic inflammation, chronic cutaneous ulceration, human papilloma virus (HPV) infection, and immunosuppression [2]. The prognosis in the metastatic disease with current treatments is generally poor and there is a need for biomarkers to predict the risk of recurrence and metastasis of primary cSCC [3]. In addition, new therapeutic strategies for the metastatic, locally advanced, and unresectable recurrent cases are in need.

A typical molecular feature in cSCC is a large mutational burden due to long-term exposure to UV radiation. Accordingly, cSCC is one of the cancers with the highest mutation rates representing a specific mutation signature [4,5,6,7,8,9]. An important early event in cSCC progression is inactivation of tumor suppressor protein p53 (*TP53*), which is followed by accumulation of additional UV-induced mutations [7]. To date, several other driver gene and tumor suppressor mutations have also been identified in cSCC [4,5,6,7,8,9]. However, these mutations can also be found with high frequency in normal epidermal keratinocytes in chronically sun-exposed skin [10]. It is, therefore, conceivable, that other factors, such as changes in the microenvironment of the keratinocytes harboring the driver mutations, are required for the progression of premalignant lesions to become invasive and metastatic cSCC [11].

The complement system consists of over 50 plasma and membrane-associated proteins, which play an important role in host defense against microbial pathogens, and in tissue homeostasis. Complement activation takes place in the tumor microenvironment as a defense mechanism against tumor cells [12]. The complement system is activated via classical, lectin, or alternative pathways, which all converge to cleavage of central complement component C3. This results in subsequent activation of the lytic pathway and formation of the membrane attack complex (MAC) on the target cell membrane, which results in the lysis of the target cell [13]. The complement system has been shown to play a role in cancer growth by promoting tumor growth, angiogenesis, and antitumor immunity [14]. Complement molecules, such as C3, have also been associated with the initiation of metastasis by contributing to epithelial-to-mesenchymal transition of tumor cells in primary tumors [15]. Anaphylatoxins produced by complement activation increase vascular permeability and, in this way, increase metastatic potential of cancer cells [16]. In this review, the role of the complement system components and inhibitors as biomarkers for advanced disease and, as potential new therapeutic targets for cSCC, will be discussed.

## 2. Cutaneous Squamous Cell Carcinoma (cSCC)

### 2.1. Epidemiology, Clinical Presentation, and Risk Factors of cSCC

Cutaneous squamous cell carcinoma (cSCC) is a keratinocyte derived carcinoma with increasing incidence worldwide [17,18,19]. It is the most common skin cancer with metastatic potential and it is regarded as the second most common skin cancer after keratinocyte-derived basal cell carcinoma [1,20]. The overall metastasis rate of primary cSCC is approximately 1% to 4% and the risk is higher in organ transplant recipients and immunosuppressed individuals [1,17,20,21,22,23]. It has been estimated that cSCC accounts for 20% of skin cancer-related mortality [1,17,21,24]. The prognosis of patients with metastatic cSCC is generally poor [23,24,25]. Mortality is associated predominantly with regional and nodal metastases instead of distant metastases [22]. In addition, the overall risk of death from any cause and the risk for second primary cancer is increased in patients with cSCC [26].

The progression of cSCC takes place from premalignant lesion, actinic keratosis (AK) to in situ cSCC (cSCCIS, Bowen’s disease) and, eventually, to invasive cSCC. The cSCC lesions typically develop in sun-exposed skin, most frequently in the head and neck region. Cumulative solar UV radiation is the primary risk factor for cSCC [27,28] and additional risk factors include male sex, advanced age, fair skin, immunosuppression [18,20,29], chronic cutaneous ulceration, HPV infection, smoking, chronic lymphocytic leukemia, non-Hodgkin lymphoma, BRAF inhibitor medication, chronic cutaneous inflammation, and hereditary blistering skin disorders, such as recessive dystrophic epidermolysis bullosa (RDEB) [1,18,25,30,31,32]. The majority of patients with cSCC present with several concurrent AK lesions and, on the other hand, only a small percentage of these precursor lesions develop into invasive cSCC. Both the risk of developing another primary cSCC and nodal metastasis increase significantly with the number of current and prior cSCC lesions [33].

Clinical presentation of cSCC varies greatly from exophytic tumors to smooth plaques and ulcerative or indurated lesions. The diagnosis of cSCC relies predominantly on histopathological examination of the lesional biopsy [34]. Metastases occur predominantly in locoregional lymph nodes, and risk factors for metastasis include a primary tumor diameter over 20 mm and tumor invasion beyond subcutaneous fat [35,36]. Other proposed risk factors associated with local recurrence and metastasis include perineural invasion, lympho-vascular invasion, poor histologic differentiation, certain histologic subtypes such as desmoplastic SCC, previous recurrence, increasing number of cSCCs, immunosuppression, chronic lymphocytic leukemia, and non-Hodgkin’s lymphoma [30,31,34,35,37]. In addition, cSCC arising in location conventionally not exposed to sunlight, such as soles of the feet or perineum, appears to show higher risk of metastasis [38].

The treatment of choice for primary cSCC is surgical excision with sufficiently wide margins. Most disease-specific deaths are preceded by local recurrence, which elevates the rate of metastasis to 25%–45%, which emphasizes the importance of precise control of margins of primary tumor excision [24,36,39]. Primary radiation therapy is an option if the patient is not eligible for surgery. If curative resection is not possible, as in an advanced and surgically inoperable or metastatic case, resection should be combined with another treatment, e.g., postoperative radiation therapy. Adjuvant radiation therapy should also be considered in cases with a high risk for local recurrence [40]. In order to detect subclinical nodal metastases, the sentinel lymph node biopsy should be considered in intermediate or high-risk cases [36].

In metastatic disease, adjuvant chemoradiation appears to offer better recurrence-free survival than radiation therapy alone [41]. Treatment options for metastatic disease are, however, limited and unestablished. Traditionally, EGFR-inhibitor cetuximab, platinum-based chemotherapeutics, and fluorouracil in variable combinations have been the most commonly drugs used off-label, but remission rates and an adverse event profile are challenges. Recently, the immune checkpoint inhibitor and the programmed cell death protein-1 (PD-1) blocking monoclonal antibody cemiplimab has been approved by FDA for treating patients with metastatic or locally advanced cSCC, who are not candidates for curative surgery or curative radiation therapy [42].

### 2.2. Carcinogenesis and Molecular Alterations in cSCC

The molecular basis for progression of cSCC is not fully understood at present. A typical feature discovered by exome sequencing of cSCCs is a large mutational burden, since, on average, 50 mutations/mega-base pair DNA have been detected in these tumors [43,44]. Accordingly, cSCC is one of the cancers with the highest mutation rate [7]. A specific UV-induced mutation pattern C>T and CC>TT predominates in cSCC (COSMIC signature 7) [5,6]. An early event in the development of cSCC is mutational inactivation of *TP53* in epidermal keratinocytes, which is shown to be mutated in up to 90% of cSCCs [4,7]. p53 plays an important role in maintaining genomic stability and its inactivation results in marked accumulation of UV-induced simple mutations [7]. Mutational activation or a high-level amplification of epidermal growth factor receptor (EGFR) have also been detected in cSCC and these downregulate the expression of p53 and Notch1 [8,45]. Additional driver mutations detected in cSCC include inactivation of *NOTCH1*, *NOTCH2*, *TGFBR1*, and *TGFBR2*, and activation of *PIK3CA* and *HRAS* [8,9,46]. Mutation and inactivation of *NOTCH1* and *NOTCH2* genes as an early event has been demonstrated in up to 85% of cSCCs, which demonstrates the tumor suppressor function of the Notch signaling pathway in keratinocytes [6]. Activating mutations in the Ras/RTK/PI3K pathway have been detected in 45% of cSCC samples in correlation with poor progression-free survival [8]. Additionally, enrichment of mutations in TGF-β, Notch, and PI3K-Akt signaling pathways has been detected in poorly differentiated cSCCs, as compared to moderately differentiated tumors [6].

Similar driver gene mutations can also be found in normal epidermal keratinocytes in chronically sun-exposed skin [10]. It is, therefore, conceivable that other factors, like changes in noncoding RNAs and the microenvironment of premalignant lesions, are required for development of AKs to invasive and metastatic cSCC [11]. For example, loss of collagen XV and collagen XVIII in the basement membrane has been demonstrated at an early stage of cSCC progression, whereas, in the later stages in invasive cSCC, collagen XV accumulates in cSCC stroma and collagen XVIII is produced by tumor cells [47]. Additionally, type VII collagen, which is a component of anchoring fibrils, has been shown to suppress vascularization of cSCC by regulating the transforming growth factor-β (TGF-β) signaling [48].

## 3. Complement System

### 3.1. Complement Activation

The complement system is a part of the innate immune system and serves as the first line of host defense against microbial pathogens. Moreover, the complement system enhances humoral immune responses and has a role in the adaptive immune system [49,50]. The complement system comprises over 50 protein components located on cell surfaces, in plasma, and in lower concentrations in the other body fluids. The main function of the complement is in the host defense against the microbial invasion and in destroying foreign structures. The complement system also recognizes damage associated molecular patterns (DAMPs) of apoptotic, damaged, or altered host cells. For example, the C1 complex promotes the clearance of immune complexes from circulation and tissues [51]. Certain complement components, such as complement factor B (CFB), complement factor D (CFD), C1r, C1s, C2, C3, C4, C5, mannose-binding lectin (MBL) -associated serine proteinases (MASP)-1, -2, and -3, and complement factor I (CFI), are serine proteinases and have functions beyond complement activation [49,52,53,54,55,56]. Liver is the primary source of the complement components, but cells in various other tissues, including epidermal keratinocytes, are able to produce complement proteins [57].

**Figure 1:**
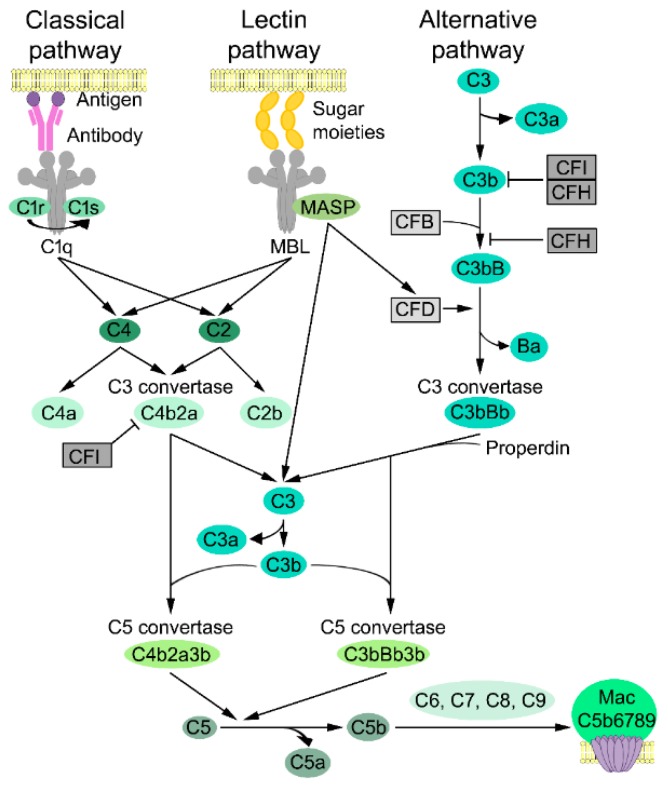
Complement cascade. The complement system can be activated via three major pathways. (1) The classical pathway is typically activated by antigen-antibody complexes on the cell surface. The C1 complex consists of C1q, C1r, and C1s molecules, and cleaves serum proteins C4 and C2. C4b binds to the target cell surface and C2a binds C4b and forms the complex C4b2a, which is a classical pathway C3 convertase. (2) The lectin pathway is activated by binding pattern-recognizing mannose-binding lectins (MBLs) to carbohydrate ligands on the surface of pathogens. Activated MBL-associated serine proteinase (MASP)-2 in the MBL-MASP-2 complex cleaves C4 and C2. The MASP-1/3 can activate complement factor D (CFD). (3) The alternative pathway is activated by any permissive surface. Constant spontaneous breakdown of C3 takes place at a low level in plasma and by the surfaces of microbes and generates C3b. Complement factor B (CFB) binds to C3b and forms a complex C3bB. Complement factor D (CFD) cleaves CFB to Ba and Bb. The fragment Bb stays attached to C3b and C3 convertase (C3bBb) is formed. Properdin stabilizes C3 convertase. All these three pathways lead to activation of the lytic pathway. C3b associates with C3 convertase to form C5 convertase and cleaves C5. C5b engages C6, C7, C8, and C9 to form the terminal membrane attack complex (MAC), which induces cell lysis. The activation of complement is strictly regulated to prevent damage to host cells. Complement factor I (CFI) and complement factor H (CFH) are the main inhibitors of complement pathways. CFH competes with CFB for binding to active C3b and inhibits C3 convertase by displacing Bb from this complex. CFI cleaves C3b to an inactive form and also inhibits the classical pathway by cleaving C4b. CFH also acts as a cofactor CFI.

Activation of the complement cascade can be initiated by interacting with a foreign target structure, which results in activation of the complement proteins in a sequential manner. This generates an extensive amplification loop. The complement cascade can be activated via three distinct pathways, which are classical, lectin, or alternative pathways. This all leads to activation of the central component C3 and, lastly, activation of the lytic pathway (Figure 1). There are three main biological responses to complement activation. First, the final step of the terminal lytic pathway leads to formation of a pore-like structure, which is a membrane attack complex (MAC), on the target cell surface. Second, the fragments of C3 activation, namely C3b and iC3b, can induce complement dependent cellular cytotoxicity (CDCC) by serving as cell surface receptors for phagocytes, such as neutrophils and macrophages. Third, C3, C4, and C5 activation fragments C3a, C4a, and C5a act as anaphylatoxins and chemotactic activators of leukocytes and can enhance immune responses [54,58]. Although the complement system is usually described as a linear cascade of three separate pathways, each pathway is tightly connected to other complement pathways [49].

### 3.2. Classical Pathway

Activation of the classical pathway is typically initiated by binding C1 to a complement-fixing antibody cluster of IgM or IgG bound to antigen on target cells. C1 is present in circulation as an inactive complex containing six C1q, two C1r, and two C1s subcomponents (Figure 1) [13,59]. Activation of the C1 complex is initiated by binding C1q to structures on microbial, apoptotic, and necrotic cells or to immunoglobulins and pentraxins (such as C-reactive protein) [49,59]. Binding of C1q to Fc domains of antibodies results in alteration in the tertiary structure of C1q, which causes a conformational change and autocatalytic activation of C1r. The activated serine proteinase C1r, in turn, cleaves the complement C1s proenzyme, which results in a change in conformation and activation of C1s [13]. Notably, no cleavage fragments are released upon activation of C1r and C1s. C1s subsequently activate serum proteins C4 and C2. C4 is cleaved to fragment C4a, which is an anaphylatoxin, and to fragment C4b, which is deposited on the adjacent surfaces. C2 is cleaved to a fragment C2b, and larger fragment C2a, which binds noncovalently to C4b on the target cell membrane. This forms the C4b2a complex, which is a classical pathway C3 convertase. C4b2a cleaves C3, which is the central component of the complement cascade, to C3a, and anaphylatoxin, and C3b results in the activation of the lytic pathway [59].

### 3.3. Lectin Pathway

In the lectin pathway, MBL and ficolins, which are homologous to MBL, act as pattern recognition molecules (PRM), which predominantly recognize carbohydrate patterns and bind to repetitive sugar moieties, i.e., mannose on the microbe cell surfaces [13,49]. MBL consists of six trimeric subunits and the structure is similar to C1q [59]. Each PRM of MBLs binds to MASP-1, MASP-2, and MASP-3, that bear structural analogy with C1r and C1s and form a C1-like complex [13]. C4 and C2 are cleaved and activated only by MASP-2 in the MBL-MASP-2 complex, which generates C3 convertase (C4bC2a) (Figure 1). MASP-1 cleaves C3 and C2 but not C4 [60] in order to increase the efficiency of convertase formation in the lectin pathway response once initiated [49]. MASP-1 and MASP-3 also enhances the activation of an alternative pathway by cleaving and activating CFD [59].

### 3.4. Alternative Pathway

Activation of the alternative pathway takes place by continuous spontaneous slow hydrolysis and breakdown of fluid phase C3 thioester (C3-H_2_O) in serum, which results in the formation of the C3b(H2O) complex. A soluble component of alternative pathway, CFB, binds to the C3b(H2O) complex [49]. The cascade continues by cleavage of CFB to fragments Bb and Ba by soluble serine proteinase CFD, which generates the initial alternative pathway convertase C3b(H2O)Bb. Fragment Bb functions as a serine proteinase and this initial convertase provides the source for formation of the amplification loop by cleaving more C3 to C3a and C3b, and generating C3 convertases (C3bBb), which cleave more C3. This results in the deposition of C3b on the cell surface [61]. The serine proteinase Bb can cleave complement component C5, plasminogen, and certain other proteins besides C3 [59]. The half-life of C3bBb is short, approximately 90 s, which results in a dissociation of Bb from the complex C3bBb [49]. The complement cascade includes a single positive regulator called properdin (complement factor P), which can stabilize the C3bBb complex up to 5–10 fold, and, this way, enhance accumulation of C3b on the target cell surface. This enables target cell phagocytosis via opsonization (Figure 1) [62].

The soluble cleavage fragment C3a functions both as an anaphylatoxin enhancing inflammation and also anti-inflammatory functions by inducing phagocyte chemotaxis, producing inflammatory mediators, and the degranulation of mast cells and granulocytes [54,63]. Association of C3b with C3 convertases (C3bBb or C4b2a) results in formation of C5 convertases, C3bBbC3b and C4b2aC3b, which initiate the lytic pathway by cleavage of C5 to C5a and C5b [59].

### 3.5. Lytic Pathway

The cleavage of C5 by C5 convertase initiates the lytic pathway, which contains complement components C5, C6, C7, C8, and C9 (Figure 1). C5 convertases, C3bBbC3b, and C4b2aC3b are generated via an alternative or a classical activation pathway, correspondingly, and are responsible for the proteolytic activation of C5 and the generation of biologically-active fragments C5a and C5b [13]. C5a is a potent proinflammatory factor and an anaphylatoxin due to its histamine-releasing ability [49,63]. C5b, which is the larger proteolytic fragment of C5. This, in turn, initiates the formation of MAC by first binding C6 and then C7. This complex, C5b67, promptly adheres to the cell surface. Binding of C8 then induces insertion of several C9 molecules to the complex, which leads to the generation of MAC (C5b6789 complex). This is then deposited on the cell surface and, eventually, leads to cell lysis.

### 3.6. Complement Inhibitors

Proper function of the complement system requires strict control of the activity of soluble and membrane-bound complement system components in order to prevent excessive component consumption and to protect host cells from complement-dependent cytotoxicity (CDC) [59]. On the host cell surfaces the C3b and C4b, fragments are inactivated by complement receptor 1 (CR1, CD35) and membrane cofactor protein (MCP, CD46). Moreover, C3 convertases are cleaved by third cell surface bound inhibitor, which is a decay accelerating factor (DAF/CD55) [61]. The lytic pathway is inactivated by membrane-bound regulator protectin (CD59). This binds C8 and C9 and prevents C9 polymerization into cell membrane lipid bilayers [49].

Several soluble inhibitors of the complement system are present in human serum. The most important inhibitor is CFI, which inhibits classical, lectin, and alternative pathways. CFI is a serine proteinase with highly restricted substrate specificity toward C3b and C4b, and it requires a cofactor to function properly. There are several cofactors for CFI e.g., complement factor H (CFH), CR1, C4b binding protein (C4bp), and MCP, which cleave C3b and C4b in different sites (Figure 1) [13].

CFH is the main soluble regulator of the alternative pathway. In addition to functioning as a cofactor for CFI in a C3b cleavage, CFH competes with CFB to the binding of C3b and displaces Bb from the C3 convertase complex (Figure 1) [49]. Serum protein C1 inhibitor (C1 INH) inhibits activation of classical and lectin pathways by inhibiting the activity of C1r, C1s, and MASP-2 [64]. C4 binding protein (C4BP) inhibits the activity of the classical pathway C3 convertase [4]. The activation of the lytic pathway is inhibited by soluble regulators clusterin (SP40) [65] and vitronectin (S-protein) [59], which bind to the forming C5b6789 complex and by complement regulatory protein CD59, which eliminates MAC from the cell surface and inhibits cytolysis [66].

## 4. Complement System in cSCC

### 4.1. Complement Components in cSCC

The majority of the epidermal layer of skin consists of keratinocytes. Epidermal keratinocytes are able to produce complement system components and inhibitors, such as C3, C4, CFB, CFH, and CFI and receptors CR1, cC1qR, C5aR1, CR2, MCP, DAF, and CD59, which have a role in host defense in skin against microbial pathogens [67,68,69,70,71]. Activation of the complement takes place in chronic inflammation, which is a recognized risk factor for many cancers, and is known to enhance tumor initiation, promotion, invasion, malignant transformation, and metastasis [72,73]. Accordingly, certain chronic inflammatory skin conditions, such as lichen planus, lichen sclerosus, and lupus vulgaris bear a risk for cSCC [1,25]. Furthermore, cSCCs induced by inflammation are aggressive and carry increased risk for metastasis [74]. The tumor microenvironment is a complex network of tumor cells, inflammatory cells, extracellular matrix, activated fibroblasts, and capillary cells, which, together, regulate tumor progression and antitumor responses [50,75,76,77]. Local production of complement components is a part of the tumor microenvironment and, recently, several studies have revealed the functional roles of locally produced complement components and inhibitors in cancer progression [78,79,80,81,82]. Alterations in the tumor microenvironment play an important role in progression of premalignant AK lesions to cSCC [11,47,48,83]. Recently, an elevated expression of several complement components and inhibitors by cSCC cells has been shown by using microarray and RNA-sequencing based expression profiling of cSCC cells and normal human epidermal keratinocytes [84,85]. The expression of components and inhibitors of the complement system by tumor cells in cSCCs in vivo has also been reported and the expression has been shown to correlate with tumor progression from AK to cSCCIS and, lastly, to invasive cSCC [84,85,86,87]. The mechanistic role of these tumor cell-derived complement components in the development of cSCC is discussed below in the following sections.

### 4.2. Classical Pathway in cSCC

**Figure 2:**
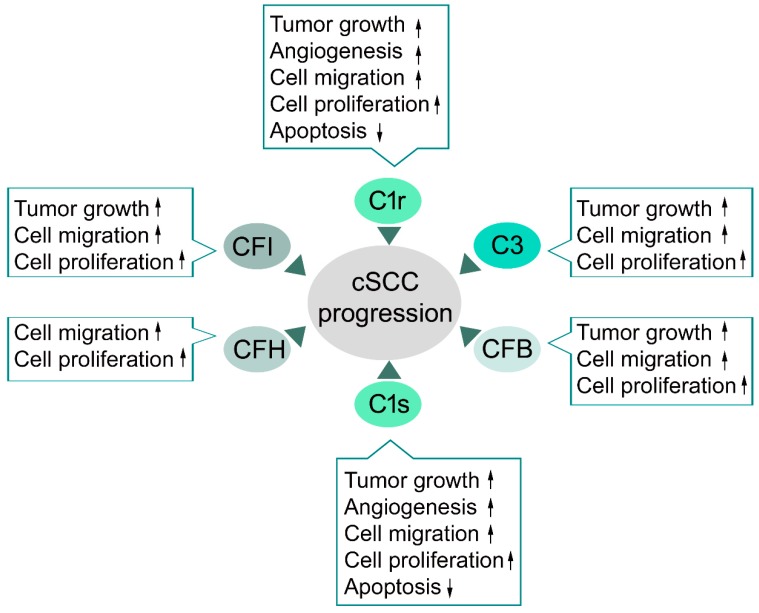
The role of tumor cell-derived complement components and inhibitors in cutaneous squamous cell carcinoma (cSCC). During progression of the cSCC complement factor H (CFH) and serine proteinases C1r, C1s, C3, CFB, and CFI exert effects on cSCC cells other than those related to complement activation. C1r, C1s, C3, CFB, and CFI promote cSCC tumor growth in vivo. C1r and C1s promote angiogenesis in cSCC tumors in vivo. C1r, C1s, C3, CFB, CFI, and CFH increase proliferation and viability of cSCC cells. C1r and C1s inhibit apoptosis of cSCC cells. The migration of cSCC cells is stimulated by C3, CFB, CFI, CFH, C1r, and C1s. ↑ indicates stimulation, ↓ indicates inhibition.

Elevated expression of classical pathway components C1r and C1s has been detected in cSCC tumor cells in a culture [84,85,87] and the expression levels of C1r and C1s determined with immunohistochemistry have been shown to correlate with tumor progression from AKs to cSCCIS and cSCC in vivo [87]. Moreover, the immunohistochemical staining intensity for both C1r and C1s was stronger in an aggressive form of cSCC from patients with RDEB (RDEBSCC) [87]. Knockdown of C1r and C1s inhibited proliferation and migration of cSCC cells and promoted apoptosis (Figure 2). Furthermore, knockdown of C1r and C1s suppresses growth and vascularization of cSCC xenograft tumors, and promotes apoptosis of tumor cells in vivo [87]. The knockdown of C1s potently inhibits the activity of ERK1/2 and PI3 kinase signaling pathways, which provides mechanistic evidence for the role of C1s in promoting proliferation and viability of cSCC cells [87]. The tumorigenic role of the classical pathway component C1q in promoting tumor progression, angiogenesis, and metastasis has been demonstrated in the murine melanoma model without the co-expression of C4, which is its downstream effector in a classical pathway [88,89]. In contrast, cSCC tumor cells do not express C1q subunits, or C4 or C2 [84,85,87]. However, it is possible, that C1q derived from circulation is present in the tumor microenvironment of cSCC tumors in vivo, which allows formation of the C1 complex. However, proteolytic activation of C1s was detected in cSCC cell cultures in the absence of C1q. Therefore, C1r and C1s can promote cSCC tumor progression independently of the activation of the classical pathway [87].

### 4.3. Alternative Pathway in cSCC

Overexpression of the alternative pathway component CFB has been detected in cSCC cells in the culture [84,85,86]. Knockdown of CFB inhibits proliferation and migration of cSCC and, significantly, inhibits growth of human cSCC xenograft tumors [86]. Knockdown of CFB in cSCC cells also potently inhibits activation of ERK1/2, which provides mechanistic evidence for the role of CFB in stimulating proliferation and viability of cSCC cells (Figure 2). Increased expression of CFB by tumor cells in cSCC in vivo was detected and there was a correlation between the expression of CFB and tumor progression and aggressiveness in cSCCs in vivo. The staining intensity for CFB is stronger in RDEBSCCs, an aggressive form of cSCC, than in UV-induced cSCCs, cSCCISs, AKs, or normal skin. In addition, the staining intensity was stronger in cSCCs than in AKs or cSCCISs [86]. These results provide evidence that CFB can promote cSCC progression by enhancing proliferation and motility of the tumor cells (Figure 2).

### 4.4. C3 and Terminal Pathway in cSCC

Elevated expression of the central complement component C3 has been detected in cSCC cells in culture, as compared to normal human epidermal keratinocytes [84,85,86]. Overexpression of C3 by tumor cells in cSCC in vivo has also been noted and stronger staining intensity for C3 was noted in RDEBSCC. In addition, the staining intensity was stronger in cSCCs than in cSCCIS or AKs [86]. Knockdown of C3 inhibited migration of cSCC cells and the growth of human cSCC xenograft tumors [86]. The expression and activation of C3 by ras-transformed HaCaT cell lines was found to correlate with the aggressive behavior of the cell lines. Intracellular activation of complement C3 by cathepsin-L has also been noted [90]. Whether intracellular activation of C3 contributes to the effect of tumor cell-derived C3 on cSCC cells remains to be elucidated.

The activation products of C3 and C5, anaphylatoxins C3a and C5a, can stimulate chemotaxis and generate radical oxygen species, which increase vascular permeability, histamine release, smooth muscle contraction, and increase blood supply and nutrition for the tissue [13,81,91]. Recently, cellular receptors for activation products of C3 and C5, i.e., C3aR and C5aR, respectively, have been shown to mediate pro-tumorigenic effects independently of their immunomodulatory functions [92]. The interaction between C5a and C5aR promotes tumor cell motility and invasion by stimulating production of matrix metalloproteinases (MMPs) [93]. Expression of anaphylatoxin receptors and production of C3 by cancer cells suggests, that anaphylatoxin receptor-mediated tumor growth is not only mediated by an immunomodulatory effect of C3, but by direct autocrine effect. This promotes tumor cell proliferation [94]. Furthermore, there is evidence that C5a promotes angiogenesis and metastasis, and that C3aR promotes dissemination of cancer cells to the central nervous system by disrupting the blood-cerebrospinal fluid barrier [92,95,96].

The lytic pathway and anaphylatoxins have been shown to promote cSCC progression in an HPV16-induced mouse model of cSCC carcinogenesis [97]. It was shown that deposition of C5a is an early feature in cutaneous squamous carcinogenesis and that urokinase-producing macrophages regulate release of C5a independently of C3 during progression of premalignant lesions. C5a, in turn, can regulate pro-tumorigenic properties of C5aR1 positive mast cells and macrophages, and suppress CD8^+^ T cell-mediated cytotoxicity. These results suggest, that C5aR1-mediated inflammation may reflect a general feature in the development of SCCs.

### 4.5. Complement Inhibitors in cSCC

The main function of the complement pathway is to destroy foreign structures in the human body. Production of complement inhibitors can protect tumor cells from CDC of the host complement system [64]. Expression of the two main soluble inhibitors of the complement system, CFI and CFH, have been detected in cSCC tumor cells in culture and in vivo and the expression correlates with the progression of cSCC and the expression in normal skin is negative or weak [84,85]. Moreover, knockdown of CFH and CFI in cSCC tumor cells results in significant inhibition of cell viability and migration in association with potent inhibition of ERK1/2 activation (Figure 2) [84,85]. Knockdown of CFI significantly suppressed the growth of cSCC xenograft tumors and promoted inflammation by increasing production of TNF-α and complement activation, as noted by the accumulation of C3b in the tumor edge [85]. These results suggest that expression of CFI and CFH by cSCC cells protects cSCC tumor cells from complement-mediated cell lysis.

In summary, expression of several complement components and inhibitors by tumor cells is detected in cSCC and they exert, in part, similar effects on progression of cSCC (Figure 2) [84,85,86,87]. CFI, C1r, C1s, and Bb, which is the active form of CFB, are serine proteinases and may cleave other substrates besides complement components. For example, C1r and C1s can activate latent MMP-9 by proposing a putative mechanism for C1s in promoting cancer angiogenesis, growth, and metastasis [98]. On the other hand, intracellular activation of C3 by cathepsin proteinases and C3-independent activation of C5 by urokinase indicate, that complement-independent activation of the complement system may play a role in progression cSCC and other cancers [97,99]. Further studies are warranted to elucidate the molecular mechanisms of the pro-tumorigenic effects of distinct complement components in cSCC.

## 5. Complement Targeted Cancer Therapy

As significant effector arm of the innate immune system, complement system plays an important role in many inflammatory conditions and has, therefore, gained attention as a potential therapeutic target in these diseases [89]. At present, numerous therapeutic compounds are at different stages of drug development both in clinical trials (Table 1) and in the preclinical phase (Table 2) [100]. It is conceivable that identification of specific complement components and inhibitors as targets in different cancers including cSCC will allow examination of the feasibility of these compounds in cancer therapy.

The lytic pathway component, anaphylatoxin C5a, is the first complement component that has emerged as a target in cancer therapy. There is an ongoing phase I/II clinical trial with anti-C5aR (IPH5401) in combination with programmed cell death ligand-1 (PD-L1) inhibitor durvalumab in patients with advanced solid cancers [101]. The concept is based on preclinical observations, that blocking C5aR results in decreased PD-L1 expression in mouse lung cancer model [102]. Accordingly, it has lately been discovered that inhibition of C5a signaling with PEGylated C5a blocking L-aptamer AON-D21 (formerly NOX-D21), in combination with PD-1/PD-L1 inhibition, inhibits tumor growth more potently than PD-1/PD-L1 inhibition alone [103].

Additional therapies targeted against complement components and inhibitors have been tested in preclinical models. C3aR antagonist SB290157 has shown efficacy against tumor growth and metastasis in mouse models of melanoma and lung cancer [104,105]. Synthetic cyclic C5aR antagonist hexapeptide AcF-(OPdChaWR) has been shown to decrease tumor growth in mouse models of ovarian and lung cancer [89,106]. Cyclic hexapeptide antagonist of C5aR1 (PMX-53) has demonstrated efficacy in immunotherapy for cSCC in combination with paclitaxel [97]. A recombinant antibody against CFH has been shown to induce a conformational change in CFH, promote complement-mediate cytoxicity on cancer cells in the culture, and inhibit tumor growth in a mouse lung cancer model [107].

Important challenges in complement targeted drug development include (1) drug dosing due to rapid turnover of complement proteins in plasma, (2) safety, and (3) non-transparency in the mechanism of addressed diseases [108]. To overcome these challenges, novel strategies have been developed. Engineered recycling antibodies are designed so that their affinity toward their target antigens changes in response to pH. They show higher affinity for their targets in neutral pH of blood (pH 7.4), which facilitates their cellular internalization through endosomes. In response to acidic pH in endosomes (pH 6), the target antigen is released and the antibody is recycled to the cell surface. Two examples of this approach are next generation C5 antibodies ravalizumab (ALXN1210) and SKY59 (RG6107/RO7112689) (Table 1) [100,108,109]. Another option is targeting the antibodies to complement neoepitopes that are exposed on complement proteins after their conformational change and activation. Examples of this strategy are C5a inhibiting antibodies ALXN1007 and IFX-1, which only target C5a neoepitope and not C5 (Table 1) [100,108,109]. In terms of safety, systemic inhibition of the complement system may predispose the patient to infections, and, therefore, localized delivery of complement targeted drugs can help reduce the safety concerns, help with dose reduction, and improve the efficacy of the drug. Mirococept (APT070) is an example of this strategy (Table 1) [100,108,109]. Structure-based drug design has been employed to design compounds including small molecules, peptides, and proteins, which target crucial interaction sites on proteins. Examples of this strategy are small molecule inhibitors of CFB, CFD (ACH-4471), and next generation C5 inhibitor peptide RA101495 (Table 1) [100,108,109,110]. In addition, CFB inhibitor IONIS-FB-LRx, and next generation C5 inhibitors zimura (ARC1905) and cemdisiran (ALN-CC5) are examples of drugs that knock down the expression of specific complement components with antisense oligonucleotides and siRNAs (Table 1) [100,108,109,110].

## 6. Conclusions

The complement system is a complex network of effectors, receptors, and regulators with many different roles. Complement components and inhibitors can exert different functions beyond complement activation. The activated complement system may enhance the evasion of tumor cells from immune responses, induce angiogenesis, promote proliferation, migration, and invasion of cSCC tumors. Complement components expressed by tumor cells can serve as biomarkers in cancer diagnosis, classification, and prognostication. A combination of complement components could be used as biomarkers for predicting aggressiveness and risk of metastasis of cSCC. As an example, a multiplex detection array of complement activation has been designed for oral SCC to assess tumor progression [155]. A similar multiplex detection array could be generated for cSCC in the future. Novel therapeutic strategies are required for treatment of advanced and metastatic cSCC. Elucidation of the expression of different complement components and inhibitors and their specific roles in progression of cSCC will allow for the identification of new therapeutic targets for treating cSCC. Complement system components could serve as potential targets in personalized cancer therapy alone or in combination with other therapies.

## Figures and Tables

**Table 1:** Complement-targeted therapeutic compounds that have entered clinical trials.

Target	Drug (Company)	Entity	Approved Indication	Clinical Trial Phase [ID] [Ref]	Clinical Trial Indication [Ref]
C1r, C1s, MASPs	C1 inhibitor (Shire)	Protein	HAE	I [NCT02435732] [100] III [NCT02547220] [100]	Renal transplantation [100]
	C1 inhibitor (Sanquin)	Protein	HAE	II [NCT02251041] [100] III [NCT01275976] [100]	Liver transplantation [100] Trauma, Sepsis, Multiple organ dysfunction syndrome [100]
	C1 inhibitor (CSL Behring)	Protein	HAE	I and II [NCT02134314] [100] II [NCT02936479] [100]	Renal transplantation [100]
	Conestat alpha (rhC1 inhibitor) (Pharming)	Protein	HAE	II [NCT02869347] [100]	Contrast-induced nephropathy [100]
C1s	BIVV009/TNT009 (Bioverativ)	Antibody	N/A	I [NCT02502903] [100]	BP, CAD, WAIHA, ESRD [100,111]
C1q	ANX007 (Annexon)	Antibody	N/A	I [NCT03488550] [112]	Open-angle glaucoma [112]
	ANX005 (Annexon)	Antibody	N/A	I [NCT03010046] [113]	Neurodegenerative and autoimmune diseases [113]
MASP-2 ^1^	OMS721 (Omeros)	Antibody	N/A	II [NCT02222545] [114] II [NCT02682407] [115] III [NCT03205995] [116]	Thrombotic microangiopathies [114] IgA nephropathy, LN, MN, C3G [115] aHUS [116]
C3	APL-2 (Apellis)	Peptide	N/A	I [NCT02588833] [100] I [NCT02264639] [100]	PNH [100,117,118]
				I [NCT02461771] [100]	AMD [CNV] [100]
				II [NCT02503332] [100]	GA due to AMD [100,119,120]
				II [NCT03226678] [121] III [NCT03500549] [117] III [NCT03531255] [118] III [NCT03525613] [119] III [NCT03525600] [120]	WAIHA, CAD [121] IgA nephropathy [122]
	AMY-101/CP40 (Amyndas)	Peptide	N/A	I [NCT03316521] [100] II [ NCT03694444] [123]	Complement-mediated diseases [100] Periodontal disease [123]
	APL-9 (Apellis)	Peptide	N/A	I [ACTRN12616000862448] [100]	PNH [100]
CFB ^1^	LNP023 (Novartis)	Small-molecule	N/A	II [NCT03439839] [124] II [NCT03373461] [125]	PNH [124] IgA nephropathy [125]
	IONIS-FB-LRx (Ionis)	Oligo-nucleotide	N/A	II [NCT03815825] [126]	AMD, GA [126]
C3 convertase	Mirococept (MRC)	Protein	N/A	II [ISRCTN49958194] [127]	Transplantation [127]
CFD ^1^	Lampalizumab (Genentech & Roche)	Antibody		II [NCT02288559] [100] III [NCT02745119] [100] III [NCT02247531] [100] III [NCT02247479] [100]	GA due to AMD [100]
	ACH-4471 (Achillion)	Small-molecule	N/A	II [NCT03053102] [128]	PNH [128]
CFP ^1^	CLG561 (Novartis)	Antibody	N/A	I [NCT01835015] [129] II [NCT02515942] {combination therapy with Tesidolumab} [100]	AMD [129] AMD, GA [100]
C5	Eculizumab (Alexion)	Antibody	PNH, aHUS, gMG	II [NCT01303952] [100]	CAD [100]
				II [NCT02093533] [100]	MPGN [100]
				II [NCT02493725] [100]	Guillain-Barre syndrome [100]
				II [NCT01567085] [100] II [NCT01919346] [100] II [NCT01895127] [100] II [NCT01399593] [100] I and II [NCT01106027] [100] II and III [NCT02145182] [100]	Renal transplantation [100]
				III [NCT02205541] [100]	STEC-HUS [100]
				III [NCT02301624] [100] III [NCT01997229] [100]	gMG [100]
				III [NCT01892345] [100]	Neuromyelitis optica [100]
	Ravulizumab/ALXN1210 (Alexion)	Antibody	N/A	I and II [NCT02598583] [100] II [NCT02605993] [100] III [NCT02946463] [130] III [NCT03056040] [131] III [NCT02949128] [132]	PNH ^1^ [100,130,131] aHUS ^1^ [132]
	Tesidolumab/LFG316 (Novartis, MorphoSys)	Antibody	N/A	I [NCT02878616] [100] II [NCT02763644] [133] II [NCT01527500] [134] II [NCT02515942] {combination therapy with CLG561} [135] II [NCT02534909] [136] II [NCT01526889] [137]	Renal transplantation [100] Transplant-associated microangiopathy [133] AMD ^1^ [134] GA ^1^ [135] PNH ^1^ [136] Uveitis [137]
	SKY59/RG6107/RO7112689 (Chugai, Roche)	Antibody	N/A	I and II [NCT03157635] [138]	PNH ^1^ [138]
	REGN3918 (Regeneron)	Antibody	N/A	I [NCT03115996] [100]	PNH ^1^ [100]
	Coversin (Akari)	Protein	N/A	II [NCT02591862] [139]	PNH ^1^ [139]
	RA101495 (Ra Pharma)	Peptide	N/A	II [NCT03030183] [140] II [NCT03078582] [141]	PNH ^1^ [140,141]
	Zimura/ARC1905 (Ophthotech)	Oligo-nucleotide	N/A	II [NCT02397954] [142] II to III [NCT02686658] [143]	IPCV ^1^ [142] AMD ^1^ [143]
	Cemdisiran */ALN-CC5 (Alnylam)	Oligo-nucleotide	N/A	I and II [NCT02352493] [144] II [NCT03303313] [145]	PNH ^1^ [144] aHUS ^1^ [145]
C5a	ALXN1007 (Alexion)	Antibody	N/A	II [NCT02245412] [146] II [NCT02128269] [147]	GVHD ^1^ [146] APS ^1^ [147]
	IFX-1 (InflaRx)	Antibody	N/A	II [NCT02246595] [148] II [NCT02866825] [149] II [NCT03001622] [150]	Sepsis, Septic shock [148] SIRS ^1^, Complex cardiac surgery [149] Hidradenitis suppurativa [150]
C5aR1 ^1^	Avacopan/CCX168 (ChemoCentryx)	Small molecule	N/A	II [NCT02464891] [151] II [NCT02384317] [152] II [NCT03301467] [153] III [NCT02994927] [154]	aHUS ^1^ [151] IgA nephropathy [152] C3G ^1^ [153] AAV ^1^ [154]
	IPH5401 (Innate Pharma)	Antibody	N/A	I [NCT03665129] {combination therapy with durvalumab} [101]	Advanced solid tumors [101]

^1^ AAV: ANCA-associated vasculitis. aHUS: Atypical hemolytic uremic syndrome. AMD: Age-related macular degeneration. AP: Alternative pathway. APS: Antiphospholipid syndrome. BP: Bullous pemphigoid. C3G: C3 glomerulopathy. CAD: Cold agglutinin disease. CFB: Complement Factor B. CFD: Complement Factor D. CFH: Complement Factor H. CFP: Complement Factor P. C5aR: C5a receptor. CNV: Choroidal neovascularization. ESRD: End-stage renal disease. GA: Geographic atrophy. gMG: Generalized myasthenia gravis. GVHD: Graft versus host disease. HAE: Hemolytic uremic syndrome. IPCV: Idiopathic polypoidal choroidal vasculopathy. LN: Lupus nephritis. MASP: Mannose-binding lectin-associated serine proteinase. MN: Membranous nephropathy. MPGN: Membranoproliferative glomerulonephritis. N/A: Not applicable. PNH: Paroxysmal nocturnal hemoglobinuria. SIRS: Systemic inflammatory response syndrome. STEC-HUS: Shiga toxin-producing E. coli-hemolytic uremic syndrome. WAIHA: Warm autoimmune hemolytic anemia. * Chemical name or description: Small-interfering RNAs (siRNAs) directed against terminal complement component 5 (C5) of the complement pathway conjugated to a *N*-acetylgalactosamine (GalNAc) ligand.

**Table 2:** Complement-targeted therapeutic molecules in the preclinical phase of drug development.

Target	Drug (Company)	Entity	Phase of Development	Indication
C1s	BIVV020 (Bioverativ)	Antibody	Preclinical [100]	CAD ^1^ [100]
C2	PRO-02 (Broteio/Argen-x)	Antibody	Preclinical [100]	Ischemia-reperfusion injury [100]
C3	AMY-103 (Amyndas)	Peptide	Preclinical [100]	Not available
C3aR ^1^	SB290157	Small molecule	Preclinical	Melanoma [104], lung cancer [105]
CFB ^1^	Bikaciomab (Novelmed)	Antibody	Preclinical [109]	AMD ^1^ [109]
C3 convertase	AMY-201/Mini-FH (Amyndas)	Protein	Preclinical [100]	Not available
CFD ^1^	CFD inhibitor (Novartis)	Small molecule	Preclinical [100]	Not available
	CFD inhibitor (Ra Pharma)	Peptide	Preclinical [100]	AMD ^1^, GA ^1^, Orphan renal diseases [100]
	ACH-5228 (Achillion)	Small molecule	Preclinical [100]	AMD, GA, C3G, IC-MPGN [100]
MASP-3 ^1^	OMS906 (Omeros)	Antibody	Preclinical [100]	AP-mediated diseases [100]
CFP ^1^	NM9401 (Novelmed)	Antibody	Preclinical [109]	PNH ^1^ [109]
CFH ^1^	5C6/Compsorbin (Amyndas)	Peptide	Preclinical [100]	Inflammation due to transplant and/or biomaterial [100]
C5	SOBI005 (Sobi)	Protein	N/A Preclinical [100]	C5-mediated diseases [100]
	C5 inhibitor (Ra Pharma)	Peptide	N/A Preclinical [100]	PNH ^1^, gMG ^1^, LN ^1^, CNS ^1^ diseases [100]
	Long-acting coversin (Akari)	Protein	N/A Preclinical [100]	Not available
	Mubodina (Adienne)	Antibody	N/A Preclinical [100]	Typical hemolytic uremic syndrome [100]
C5a	IFX-2, IFX-3 (InflaRx)	Antibody	N/A Preclinical [100]	Not available
	NOX-D19 to NOX-D21	L-RNA Aptamer (Spiegelmer)	N/A Preclinical [109]	Sepsis and transplant rejection [109]
C5aR1 ^1^	DF2593A (Dompe)	Small molecule	N/A Preclinical [100]	Inflammatory and neuropathic pain [100]
	PMX-53	Peptide	N/A Preclinical	SCC ^1^ [97]
	AcF-(OPdChaWR)	Peptide	N/A Preclinical	Cervical cancer [88]
	ALS-205 (Alsonex)	Peptide	N/A Preclinical [100]	ALS ^1^, Alzheimer disease, Huntington disease [100]

^1^ ALS: Amyotrophic lateral sclerosis. AMD: Age-related macular degeneration. AP: Alternative pathway. CAD: Cold agglutinin disease. CFB: Complement Factor B. CFD: Complement Factor D. CFH: Complement Factor H. CFP: Complement Factor P. C3G: C3 glomerulopathy. C3aR: C3a receptor. C5aR: C5a receptor. CNS: Central nervous system. GA: Geographic atrophy. gMG: Generalized myasthenia gravis. IC-MPGN: Immune-complex membranoproliferative glomerulonephritis. LN: Lupus nephritis. MASP: Mannose-binding lectin-associated serine proteinase. N/A: Not applicable. PNH: Paroxysmal nocturnal hemoglobinuria. SCC: squamous cell carcinoma.
